# Identification of regenerative processes in neonatal spinal cord injury in the opossum (*Monodelphis domestica*): A transcriptomic study

**DOI:** 10.1002/cne.24994

**Published:** 2020-08-04

**Authors:** Benjamin J. Wheaton, Johnny Sena, Anitha Sundararajan, Pooja Umale, Faye Schilkey, Robert D. Miller

**Affiliations:** ^1^ Department of Integrative Medical Biology University of Umeå Umeå Sweden; ^2^ Center for Evolutionary and Theoretical Immunology, Department of Biology University of New Mexico Albuquerque New Mexico USA; ^3^ National Center for Genome Resources Santa Fe New Mexico USA

**Keywords:** axon regeneration, GO category overrepresentation, gray short‐tailed opossum (*Monodelphis domestica)*, RNA sequencing, spinal cord injury

## Abstract

This study investigates the response to spinal cord injury in the gray short‐tailed opossum (*Monodelphis domestica*). In opossums spinal injury early in development results in spontaneous axon growth through the injury, but this regenerative potential diminishes with maturity until it is lost entirely. The mechanisms underlying this regeneration remain unknown. RNA sequencing was used to identify differential gene expression in regenerating (SCI at postnatal Day 7, P7SCI) and nonregenerating (SCI at Day 28, P28SCI) cords +1d, +3d, and +7d after complete spinal transection, compared to age‐matched controls. Genes showing significant differential expression (log2FC ≥ 1, *Padj* ≤ 0.05) were used for downstream analysis. Across all time‐points 233 genes altered expression after P7SCI, and 472 genes altered expression after P28SCI. One hundred and forty‐seven genes altered expression in both injury ages (63% of P7SCI data set). The majority of changes were gene upregulations. Gene ontology overrepresentation analysis in P7SCI gene‐sets showed significant overrepresentations only in immune‐associated categories, while P28SCI gene‐sets showed overrepresentations in these same immune categories, along with other categories such as “cell proliferation,” “cell adhesion,” and “apoptosis.” Cell‐type–association analysis suggested that, regardless of injury age, injury‐associated gene transcripts were most strongly associated with microglia and endothelial cells, with strikingly fewer astrocyte, oligodendrocyte and neuron‐related genes, the notable exception being a cluster of mostly downregulated oligodendrocyte‐associated genes in the P7SCI + 7d gene‐set. Our findings demonstrate a more complex transcriptomic response in nonregenerating cords, suggesting a strong influence of non‐neuronal cells in the outcome after injury and providing the largest survey yet of the transcriptomic changes occurring after SCI in this model.

## Graphical abstract

In opossums, spinal injury early in development results in spontaneous axon growth through the injury, but this regeneration diminishes with maturity until it is lost entirely. Analysis of the postinjury transcriptomes suggests that there is a more complex transcriptomic response in nonregenerating cords, suggesting a stronger influence of non‐neuronal cells in the outcome after injury, and suggesting that a prolonged immune response may be inhibitory to spinal cord regeneration.

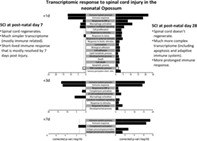

## 1 INTRODUCTION

Injuries to the adult central nervous system (CNS), especially to the spinal cord, yield a poor prognosis for recovery. Depending on the injury's severity, the resulting loss of function below the level of the injury is usually permanent and leaves the patient with debilitating disabilities and susceptible to a range of related secondary maladies. These problems, combined with the absence of an effective treatment, present a difficult outlook for spinal cord injured patients. Underlying this clinical situation is a failure of severed axons to regenerate, at least over sufficient distances or with sufficient reconnections to be of benefit.

In contrast to the adult, the immature nervous system retains a considerable ability to regenerate following injury. This has been demonstrated extensively using marsupials, whose young are born at a very immature stage of neural development, similar to an embryonic Day 13 (E13) rodent (Saunders et al., 1992; Saunders, Deal, Knott, Varga, & Nicholls, 1995).

In these models, for a short period after birth (approximately 1–3 weeks, depending on the species), injury to the spinal cord is followed by pronounced outgrowth of axons through the injury site (Fry, Stolp, Lane, Dziegielewska, & Saunders, 2003; Lane et al., 2007; Saunders et al., 1995; Wheaton, Callaway, Ek, Dziegielewska, & Saunders, 2011). This axonal outgrowth is able to span the injury site and connect the disrupted ends of the transected spinal cord as early as 5 days postinjury (Lane et al., 2007) and is composed of a combination of regenerating axons and newly growing axons, both of which are able to grow across the injury site (Fry et al., 2003; X. M. Wang, Terman, & Martin, 1998).

This robust regrowth after early‐age injury is maintained through to adulthood and has been demonstrated in the gray short‐tailed opossum (*Monodelphis domestica*; Saunders et al., 1992; Saunders et al., 1995), the North American opossum (*Didelphis virginiana*; Martin, Terman, & Wang, 2000; X. M. Wang, Terman, & Martin, 1996) and the tammar wallaby (*Macropus eugenii*; Saunders et al., 2017). In all three species it is accompanied by supraspinally mediated functional recovery of coordinated hindlimb locomotion by adulthood (Saunders et al., 1998; Saunders et al., 2017; X. M. Wang, Basso, Terman, Bresnahan, & Martin, 1998; Wheaton et al., 2011; Wheaton et al., 2013). The regenerative response diminishes if injuries are made later in development (Lane et al., 2007) until no regrowth occurs, similar to adult injuries (Wheaton et al., 2011). This unique mammalian model, in which spinal cord regrowth occurs following complete spinal transection in the absence of any external interventions has the capacity to demonstrate key processes and events that underlie successful axon regeneration.

The exact mechanisms underlying this axon regeneration remain unknown. Early work showed a positive role for cAMP in regenerating injuries (Mladinic, 2007), and that increased annexin‐1 expression was associated with the loss of regenerative capacity (Mladinic, Del Bel, & Nicholls, 2007). Proteomic studies implicated ubiquitin, among other proteins, in the response (Noor et al., 2011; Noor et al., 2013).

Few large‐scale postinjury gene analysis studies have been performed in this model. Those that havehighlighted the complexity of the responses and demonstrated that there are substantial age‐dependent differences. Microarray analysis of ex vivo opossum spinal cords maintained under culture conditions showed an increase in genes relating to cell proliferation and antiapoptotic functions in regenerating cords, and an increase in proapoptotic and cell adhesion genes in nonregenerating cords (Mladinic, Lefevre, Del Bel, Nicholls, & Digby, 2010). Using RNA sequencing of injured spinal cords in an in vivo model, Saunders et al. (2014) found that regenerating injuries only resulted in a comparatively small number of significant gene changes, almost all of which were related to immune/inflammatory functions, but in nonregenerating injuries, the genetic response involved a larger number of genes from a much wider range of categories. These studies focused on very short term changes, limiting their scope to 1 day postinjury and although many gene‐sequencing studies of spinal cord injury have been performed over longer time periods postinjury (e.g., Aimone, Leasure, Perreau, & Thallmair, 2004; K. Chen et al., 2013), none has examined the time‐course of gene regulation following injuries in the mammalian nervous system where regeneration occurs.

Therefore, in the present study we have applied an RNA‐sequencing approach to identify the gene expression profiles of regenerating and nonregenerating mammalian spinal cords in *M. domestica* and sought to characterize the injury's progression by examining the transcriptomes from multiple time‐points after injury at either age.

Our study demonstrated a dynamic and multifaceted response to injury that was more complex in nonregenerating cords than regenerating cords, possibly resulting from the relative maturity of a number of systems, including the adaptive immune system, myelination and apoptosis. At its most basic level, this study provides a list of genes that respond to spinal cord injury in cords that regenerate and those that do not and therefore may be a useful resource from which one can determine genes that play important roles in driving spinal cord regeneration.

## 2 MATERIALS AND METHODS

### 2.1 Ethics statement

All animals for experimental use were sourced from an in‐house breeding program at the Department of Biology, University of New Mexico. All *M. domestica* breeding and experimental work was conducted under protocols approved by the Institutional Animal Care and Use Committee of the University of New Mexico, protocol numbers 15‐200334‐B‐MC and 15‐200330‐HSC.

### 2.2 Spinal cord injuries and tissue collection

Complete spinal cord transections were performed on opossum pups at either postnatal Day 7 or 28 (P7SCI and P28SCI). Cords were collected for RNA sequencing analysis +1, +3 or +7 days later, along with age‐matched control tissues. Detailed procedures for completely transecting the lower thoracic (T10) spinal cord in these pups have been described previously (Wheaton et al., 2011; Wheaton et al., 2013; Wheaton, Noor, Dziegielewska, Whish, & Saunders, 2015). Briefly, opossum pups were anesthetized to a surgical level using inhaled isoflurane (3% in O_2_‐enriched air), either while still attached at the teat to the anesthetized mother (in the case of P7SCI pups) or individually (in the case of the more mature P28SCI pups). A longitudinal incision was made in the shaved skin overlying the lower thoracic spine and, using blunt dissection of the underlying musculature, the T10 vertebra was exposed. Spinal cord transection was performed by first dissecting between the vertebrae, opening the membranous intervertebral connective tissue with a fine ophthalmic scalpel (Eagle Labs, 15° stab blade) before completely transecting the spinal cord using fine spring scissors (Fine Science Tools). The point of a fine ophthalmic blade was then repeatedly passed through the lesion site in contact with the underlying bone in order to sever any remaining axons and confirm the completeness of the transection. Once the transection was complete, the skin incision was closed and sealed using surgical tissue glue (Vetbond, 3M).

Animals were maintained under surgical anesthesia on a heated pad (25–28°C) for the duration of the surgery and recovery. Postoperative pain was managed with intraperitoneal injections of buprenorphine (0.06 mg/kg). No postoperative infections were observed.

After 1, 3, or 7 days postinjury pups were killed by isoflurane overdose and decapitation. Spinal cord tissue (1 cm of cord encompassing the injury site) was collected for RNA sequencing, along with age‐matched control tissue from the same spinal levels, in RNase‐free tubes, snap frozen in liquid nitrogen and stored at −80°C until RNA extraction.

### 2.3 RNA preparation and sequencing

RNA sequencing was performed on 3–4 samples per experimental group. At some ages it was necessary to pool spinal cords in order to attain the required RNA concentration for library preparation. To produce a single biological replicate, cords from three animals were pooled for groups P7SCI + 1d, P8 control, P7SCI + 3d and P10 control; cords from two animals were pooled for groups P7SCI + 7d and P14 control. Cords from all P28SCI groups and their corresponding controls did not require pooling.

RNA was extracted using the Trizol method (Chomczynski & Sacchi, 1987) and washed, purified and eluted using the PureLink RNA Mini Kit (Ambion) according to the manufacturer's instructions. Briefly, spinal cords were manually homogenized in TRIzol reagent (Ambion) using a dounce glass tissue grinder (Wheaton) followed by chloroform extraction and centrifugation (12,000×*g*, 15 min at 4°C). The aqueous, RNA‐containing phase was retained and precipitated with 70% ethanol. The RNA in the resultant mix was bound to a spin cartridge and washed repeatedly, as per the manufacturers instructions. RNA was eluted in RNase‐free water. Quality was assessed using the Agilent 2100 Bioanalyzer (RRID:SCR_013575); all samples used for RNA sequencing had RIN > 9. Quantity was determined using a Qubit 2.0 Fluorometer.

Library preparation and RNA sequencing was performed at the National Centre for Genome Resources (NCGR, Santa Fe, NM) using the Illumina TruSeq library preparation protocol. The poly‐adenylated coding mRNA was isolated and fragmented before the strands were reverse transcribed to produce cDNA libraries. Libraries were sequenced using the HiSeq 2000 instrument (Illumina; RRID:SCR_010233). Contaminants (e.g., adaptors, dimers, etc.) were removed from the samples using NCGR's custom filtering pipeline, resulting in an average of 17.8 M single‐end 50 bp postprocessed reads generated per sample.

### 2.4 Read mapping, transcript identification, and differential expression analysis

RNA‐seq data were analyzed using the Lumenogix Bioinformatics‐in‐a‐Box platform, a web‐based bioinformatics interface offering a variety of tools (Lumenogix, NCGR, Santa Fe, NM; www.ncgr.org/; Umylny & Weisburd, 2011; National Center for Genome Resources, RRID:SCR_012416). Quality assessment was performed using FastQC (RRID:SCR_014583). Since all samples displayed reads with Phred scores greater than 25, no further quality‐based trimming was required. Two pipelines were used in parallel in the present study. Pipeline 1 invoked the tuxedo suit package of algorithms where reads were mapped to the *M. domestica* reference genome using Tophat2 (v. 2.0.11; RRID:SCR_013035) producing gene‐based FPKM (fragments per kilobase mapped reads) intensities that were quantified using Cufflinks (v. 2.2.1; RRID:SCR_014597). Differences in gene abundance were assessed with CuffDiff (v. 2.2.1; RRID:SCR_001647). In pipeline 2, reads were mapped using GSNAP (RRID:SCR_005483) which produces simple gene‐based raw hit counts, which were quantified using HTSeq (v. 0.6.0; RRID:SCR_005514). Differential gene expression was performed using EdgeR (v. 2.0.5; RRID:SCR_012802) and DESeq (v. 1.2.1; RRID:SCR_000154), differential expression analysis tools that use built‐in upperquartile and geometric mean normalization functions, respectively. All analyses were performed using default algorithm thresholds and parameters. In both pipelines the reads were mapped and quantified with reference to the *M. domestica* reference genome (assembly: MonDom5; annotation: Ensembl release 76), which yielded reads for 23,899 genes for pipeline 1 (using Tophat2) or 19,311 genes for pipeline 2 (using GSNAP).

**FIGURE 1:**
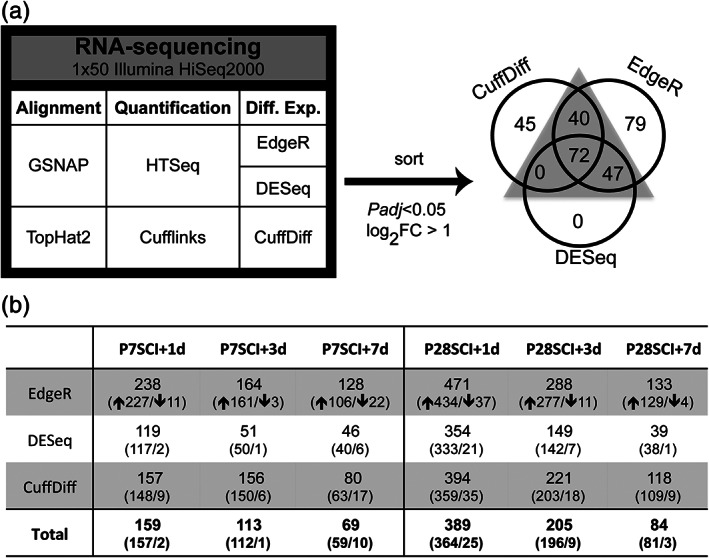
Pipeline for identifying differentially expressed genes. (a) Multiple bioinformatic algorithms were used to identify differentially regulated genes following spinal cord injury. Identified genes were sorted (*Padj* ≤ 0.05; log2FC ≥ 1 differential expression in either direction) and the resulting gene‐sets were overlaid. Genes that were identified as differentially expressed by at least two algorithms were used for downstream analysis. Venn diagram shows data for P7SCI + 1d, where genes contained in gray triangle were considered differentially expressed. (b) Summary of differentially expressed genes from all differential expression algorithms at all ages analyzed. Numbers in brackets represent the numbers of up−/down‐regulated genes. Total represents the number of genes for each age identified by at least two algorithms, taken forward into the remaining analyses

Two types of biological comparisons were performed. SCI samples at each injury age were compared with age‐matched control tissue to examine injury‐induced genes. Uninjured control tissue at P8 and P29 was also compared to examine developmentally regulated gene expression. Within each of the three algorithms, the genes whose abundance changed in response to injury or development were identified by selecting those with a *Padj* value (false discovery rate–adjusted *p* value, accounting for multiple comparisons) less than 0.05 and log2FC greater than 1 in either direction, relative to their control comparison. Next, the final gene‐set was determined by overlaying these individual lists using Venn diagrams and selecting only those genes that had been identified by two or more algorithms (see Figure 1a for graphical representation of this workflow). The validity of this somewhat conservative approach was tested by mining the developmental comparisons for neurodevelopmental genes.

### 2.5 Gene‐set overrepresentation

To determine functional gene categories that were involved in the response to spinal injury at the two ages, Gene Ontology Overrepresentation analysis was performed using the online PANTHER database (www.pantherdb.org; Mi, Muruganujan, & Thomas, 2013). This tool compares a test gene list to a reference gene list, and uses a simple binomial statistical test in order to determine whether any particular ontology class is over‐ or underrepresented in the input gene list. Here, the test gene list comprised all differentially expressed genes (both up‐ and down‐regulated) in each time‐point's data set, compared with a reference gene list encompassing all genes in the *M. domestica* genome. Overrepresentation analysis was performed for the GO terms classified in the GO Slim Biological Process subclass. GO Slim terms with *p ≤* .05 (Bonferroni correction for multiple comparisons) were considered significant, and graphed. The “unclassified” category was not shown in all analyses. Individual genes comprising each significant GO category were then extracted, and heatmaps of each gene's fold change were constructed in order to further examine the composition of the functional groups.

### 2.6 Cell‐type association analysis

To understand the likely cell source of the differentially expressed genes in this study, we used a publicly available CNS cell‐type–specific transcriptome to determine whether injury‐induced genes demonstrated any bias toward a particular cell type. Single‐cell‐type data representing transcriptomes from individual cell populations derived from mouse brain tissue was downloaded from The Barres Lab webpage (http://web.stanford.edu/group/barres_lab/brain_rnaseq.html; Y. Zhang et al., 2014). This data set provides the expression level for every gene transcribed in seven different CNS cell types: astrocytes, neurons, endothelial cells, microglia, oligodendrocyte progenitor cells (OPCs), newly formed oligodendrocytes and myelinating oligodendrocytes. Here it provided the background expression information to which *M. domestica* spinal injury–induced genes were compared. Since we were interested in the proportion of a gene's expression that could be attributed to a particular cell type, rather than its raw expression level, each gene's cellular expression level was shown as a percentage of its sum expression across all cell types. Opossum Ensembl gene codes were converted to common gene identifiers that were then used to extract the specific expression data from the cell population data set. Any gene with an identifier not found in the mouse data set was excluded along with any identified gene whose expression level did not rise above background level (FPKM = 0.1 in the background single cell type study data). For each remaining injury‐induced gene of interest the expression level in each of the cell‐types imposed from the background data set was recorded and heatmaps were constructed. Resulting data were first separated by time‐point then clustered hierarchically based on the commonality of their expression patterns in the cell types using the Morpheus tool (Broad Institute; https://software.broadinstitute.org/morpheus; RRID:SCR_017386).

### 2.7 Analysis

All bioinformatics processing and analyses were performed using the Lumenogix Bioinformatics‐in‐a‐Box web platform (Umylny & Weisburd, 2011) and GO term overrepresentation analysis was performed using the Panther database (www.pantherdb.org; Mi et al., 2013). Venn diagrams were created using the online tool GeneVenn (genevenn.sourceforge.net; RRID:SCR_012117). Heat maps were constructed and clustering was performed using the Broad Institute's Morpheus tool (https://software.broadinstitute.org/morpheus; RRID:SCR_017386), available online. For analysis of regeneration‐associated genes (RAGs) and neurodevelopmental genes, FPKM values resulting from the cufflinks package were used to assess expression levels and gene expression heatmaps were constructed in Morpheus. All graphs were produced using GraphPad Prism v5.0b (RRID:SCR_002798). All figures were produced using the Adobe Creative Suite (RRID:SCR_010279; RRID:SCR_014199).

## 3 RESULTS

### 3.1 Identifying differentially expressed genes

In order to study transcriptional changes occurring during successful regeneration we performed a large‐scale, multi‐time–point RNA sequencing study encompassing three postinjury time‐points (+1, +3, +7 days) after injuries made at P7 (where regrowth occurs) and injuries made at P28 (where no regrowth occurs). Three differential expression algorithms (EdgeR, DESeq, and CuffDiff) were used to identify genes whose abundance changed in response to spinal injury (Figure 1).

Since these algorithms each identified a slightly different subset of genes at each time‐point we employed a conservative method whereby only genes that were identified as significantly different (*Padj* ≤ 0.05; log2FC ≥ 1 in either direction) by at least two out of the three algorithms were considered for further analysis (Hansen, Schilkey, & Miller, 2016). An example of this workflow for identifying DE genes in the P7SCI + 1d data set is shown in Figure 1a.

In all time‐points studied, EdgeR consistently identified the largest number of genes, while DESeq proved to be the most conservative, identifying on average, less than half the genes compared to EdgeR (Figure 1b). In all cases this more conservative DESeq data set was nearly entirely captured by the other algorithms whereas genes identified by both EdgeR and CuffDiff contained many unique genes not identified by either of the other algorithms, which resulted in >30% of their data sets not being included in the final analysis.

**FIGURE 2:**
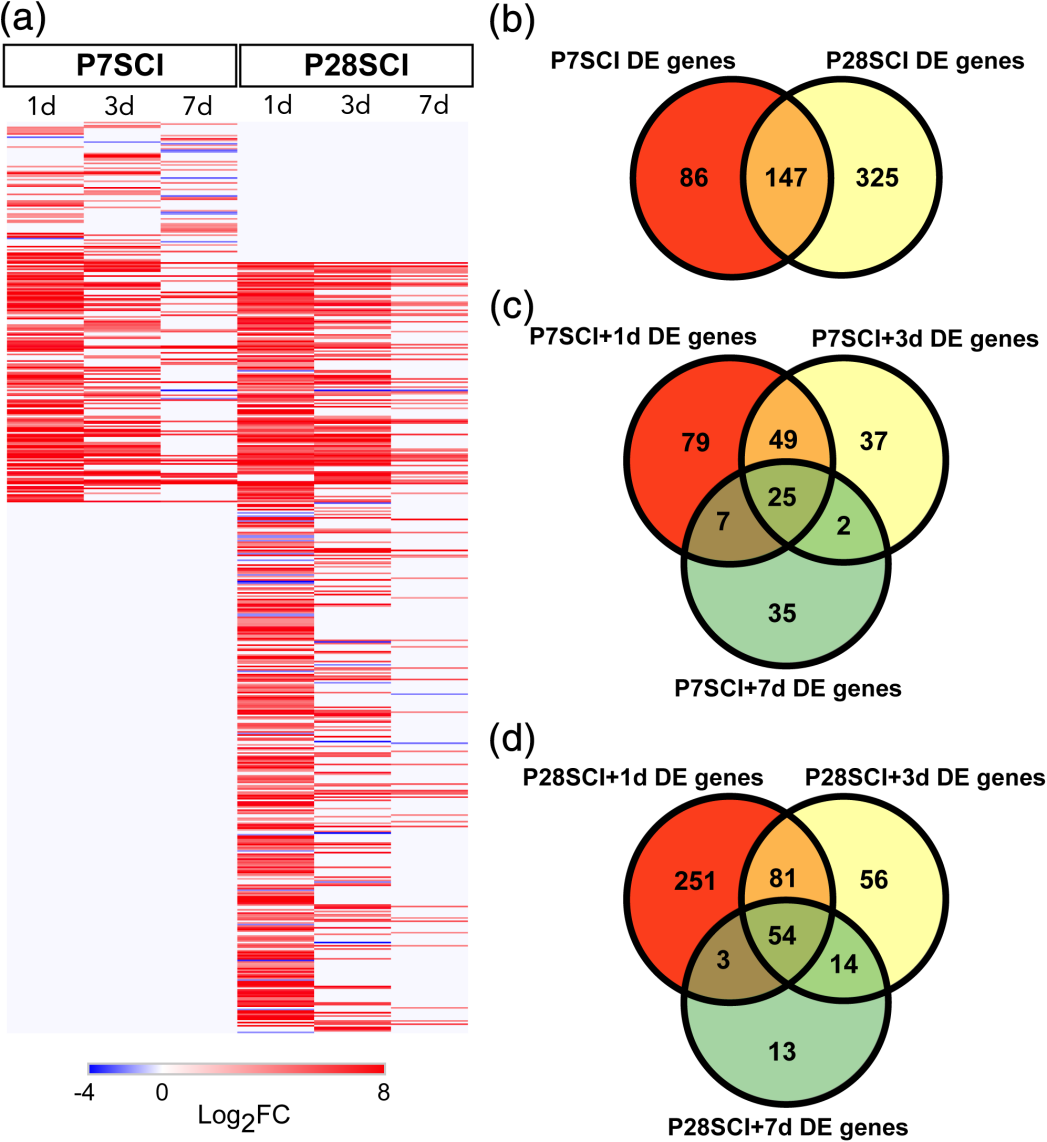
Differentially expressed genes following spinal cord injury in regenerating (P7SCI) and nonregenerating (P28SCI) spinal cords. (a) Five hundred and fifty‐eight genes were differentially regulated at one or more time‐points after injury, the majority of which were upregulated (red bars). Venn diagrams display the number of differentially expressed genes that were shared or unique to P7SCI or P28SCI (b) and those shared or unique for individual time‐points following injury at P7SCI (c) and P28SCI (d) [Color figure can be viewed at wileyonlinelibrary.com]

The transcriptomic response to spinal injury changed considerably depending on the postinjury regenerative outcome or postinjury time‐point (Figure 2). In total, injury altered the transcript abundance of 558 genes (*Padj* ≤ 0.05; log2FC ≥ 1 in either direction) at one or more time‐points after either lesion (Figure 2a), with the majority of these being upregulations relative to control. Over 60% of the genes found in the P7SCI groups were also found in P28SCI groups, demonstrating significant transcript overlap between regenerating and nonregenerating spinal cords. However, a large number of genes with changed abundance were identified uniquely in regenerating or nonregenerating cords only (Figure 2a,b). A complete list of injury‐induced differentially expressed genes and fold changes is shown in Appendix 1.

At both injury ages (P7SCI and P28SCI) the greatest number of differentially expressed genes was found acutely after injury, before decreasing as time progressed. This was evident in gene‐sets resulting from individual algorithms (as shown in Figure 1b) as well as for the combined gene‐set.

To investigate whether this was simply a turning off of the gene response, we overlaid the genes identified at each time‐point to assess common and distinct elements of the transcriptome after injury (Figure 2c,d and Appendix 1). In both injury groups a small subset of genes (approximately 10% at both injury ages) were consistently identified at all postinjury time‐points. These included, for example, the complement factors *C1S*, *C1QA*, *C1QB*, and *C1QC* and other immune‐related genes including *LBP*, *CSF2RB*, *CSF1R*, *MPEG1*, and *IL1R1*. Although the magnitude of differential expression changed with time, the direction, up‐ or down‐regulation, was identical in all cases.

The earliest time‐point (+1d postinjury) contained the greatest number of distinct genes whose abundances changed due to injury. This was a particularly prominent feature in the P28SCI group, where 251 genes, well over half of the total genes that were differentially expressed in the P28SCI group, were differentially expressed only at +1d after injury. Gene‐sets identified in the early phase (+1 and +3d) had many more elements in common than either did with the gene‐sets identified at the later phase (Figure 2c,d). Interestingly, in both injury groups, a small number of genes were differentially expressed in both +1d and +7d cords.

### 3.2 Injury‐associated gene function

**FIGURE 3:**
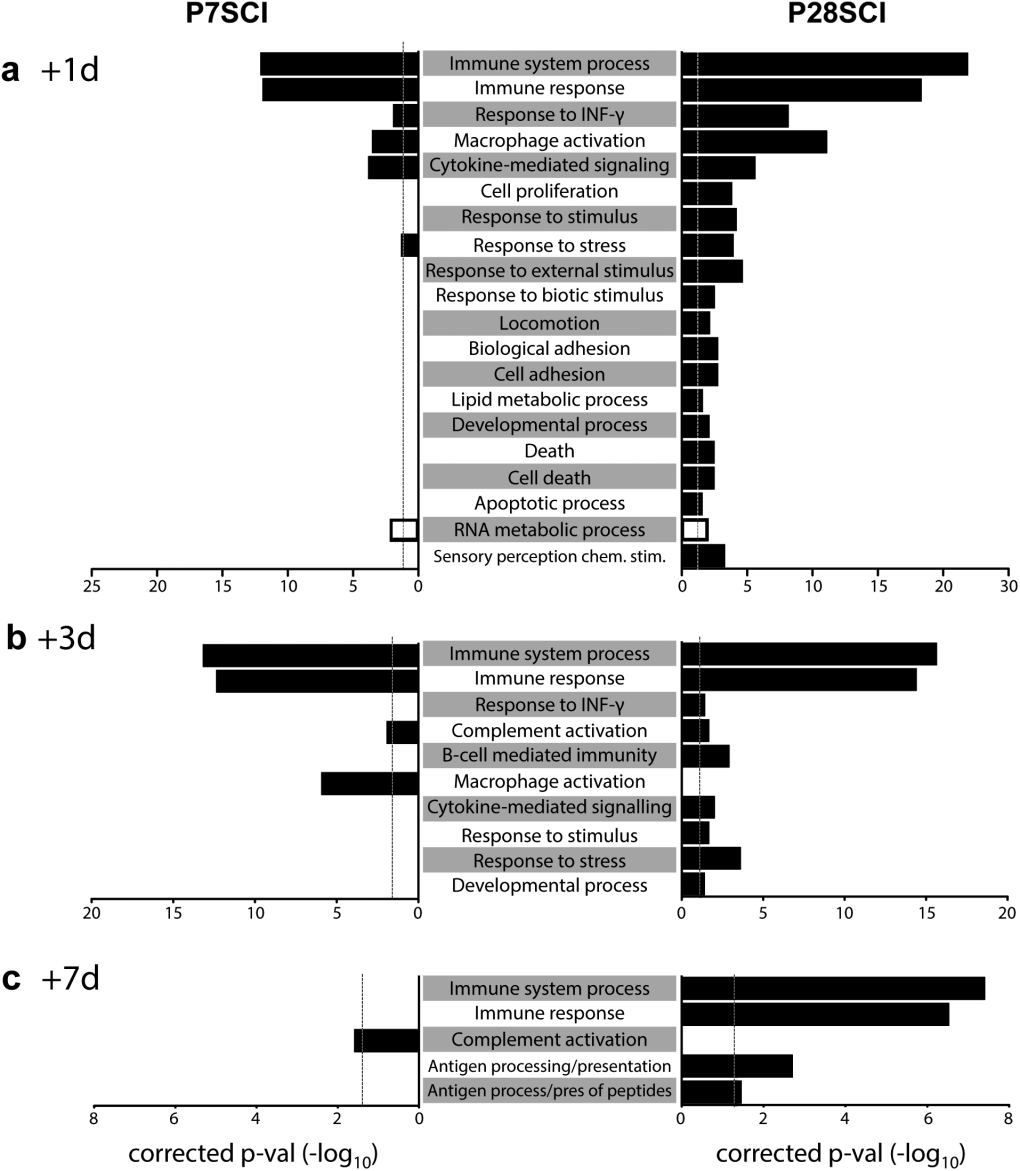
Functional classification of injury‐induced genes. Statistically significant overrepresented (black bars) and underrepresented (white bars) gene ontology categories for P7SCI (left sides) and P28SCI (right sides) are shown for +1d (a), +3d (b), and +7d (c) after injury. The –log10 corrected *p*‐value for overrepresentation was plotted for all significant categories. Dashed line represents *p* = .05, log transformed. *Note*: For graphical comparative purposes, the P7SCI has been flipped; however, these do not denote downregulations or underrepresentations

In order to characterize the genes that are regulated after injury we sought to classify them according to functional category. The genes identified at each time‐point were used for overrepresentation analysis of GO Slim categories in the Biological Process parent category (Figure 3).

Apparent is that injury at P28 elicited a much more complex array of response categories than at P7. Gene categories overrepresented in the P7SCI data were limited almost entirely to immune‐related functions at all ages studied (e.g., “Immune system process,” “Macrophage activation,” “Cytokine‐mediated Signaling,” “Complement activation”). These immune categories were also overrepresented at P28SCI, particularly early after injury. However, categories such as “Cell proliferation,” “Cell locomotion,” “Cell adhesion,” “Death,” “Cell Death,” and “Apoptosis” were also overrepresented in the P28SCI data (Figure 3a). The acute time‐point was also the only time at which an underrepresentation of a category was observed, wherein genes relating to “RNA‐metabolic process” were significantly underrepresented in both +1d injury groups (Figure 3a). As time progressed after either injury, and as the number of differentially expressed genes decreased, the number of overrepresented categories became more simplified and by +7d postinjury, only immune‐related categories remained overrepresented (Figure 3a). Interestingly in this latest time‐point no common overrepresented categories were identified in both P7SCI and P28SCI data (i.e., only “Complement Activation” was significantly overrepresented at P7SCI + 7d whereas “Immune system process,” “Immune response,” “Antigen processing and presentation,” and “Antigen processing and presentation of peptides” were overrepresented in P28SCI + 7d data; Figure 3c).

**FIGURE 4:**
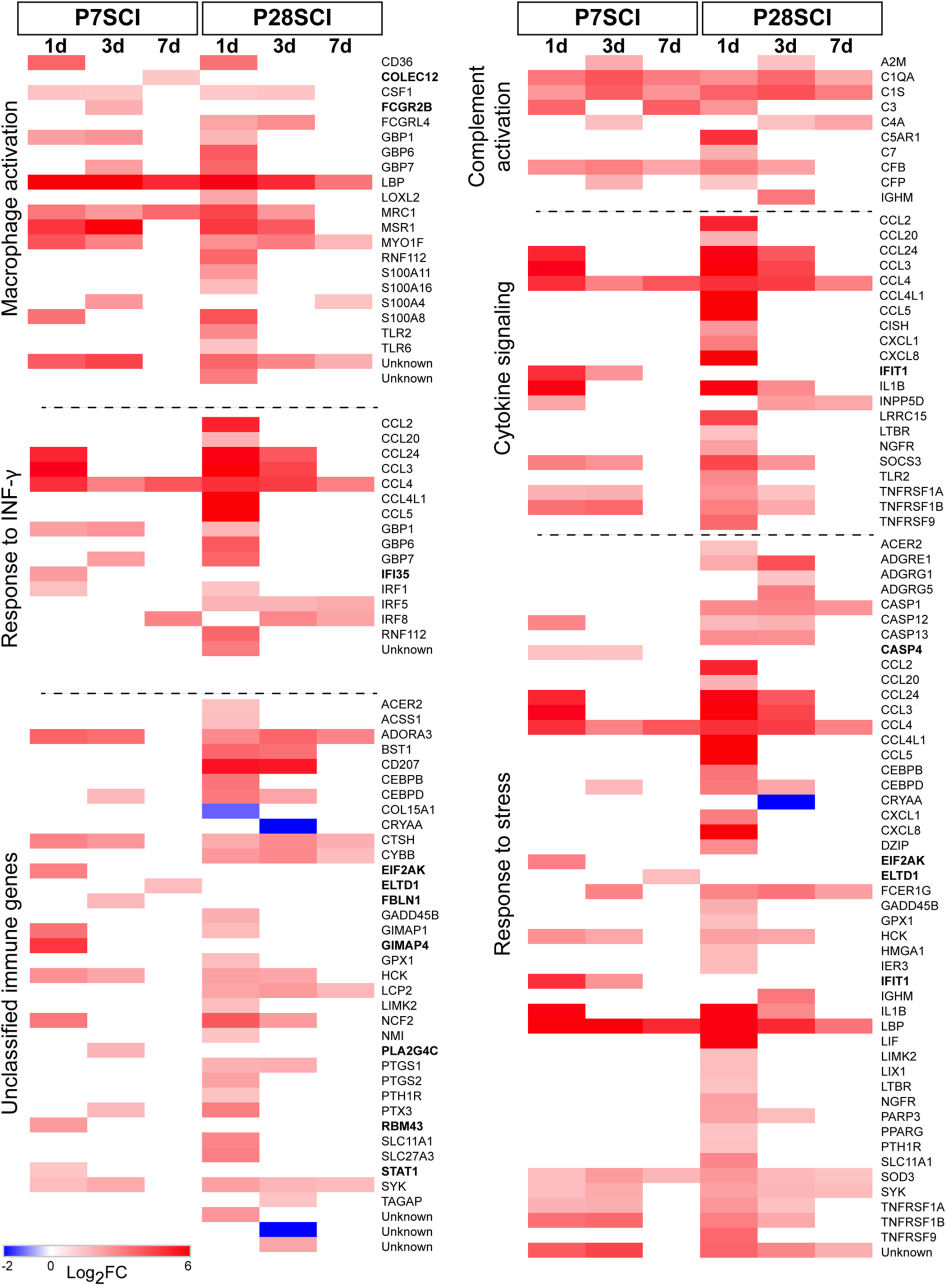
Individual genes classified in key functional categories in regenerating and nonregenerating injury groups. The genes that were classified in GO slim categories that were significantly overrepresented at one or more time‐points after injury in both P7SCI and P28SCI animals are shown along with their fold changes. The “Unclassified immune genes” category contains genes appearing in “Immune system process” category but not classified in any of its child categories. Bold gene names indicate those that only appear in P7SCI data sets. *Note*: Some genes are classified in multiple groups [Color figure can be viewed at wileyonlinelibrary.com]

**FIGURE 5:**
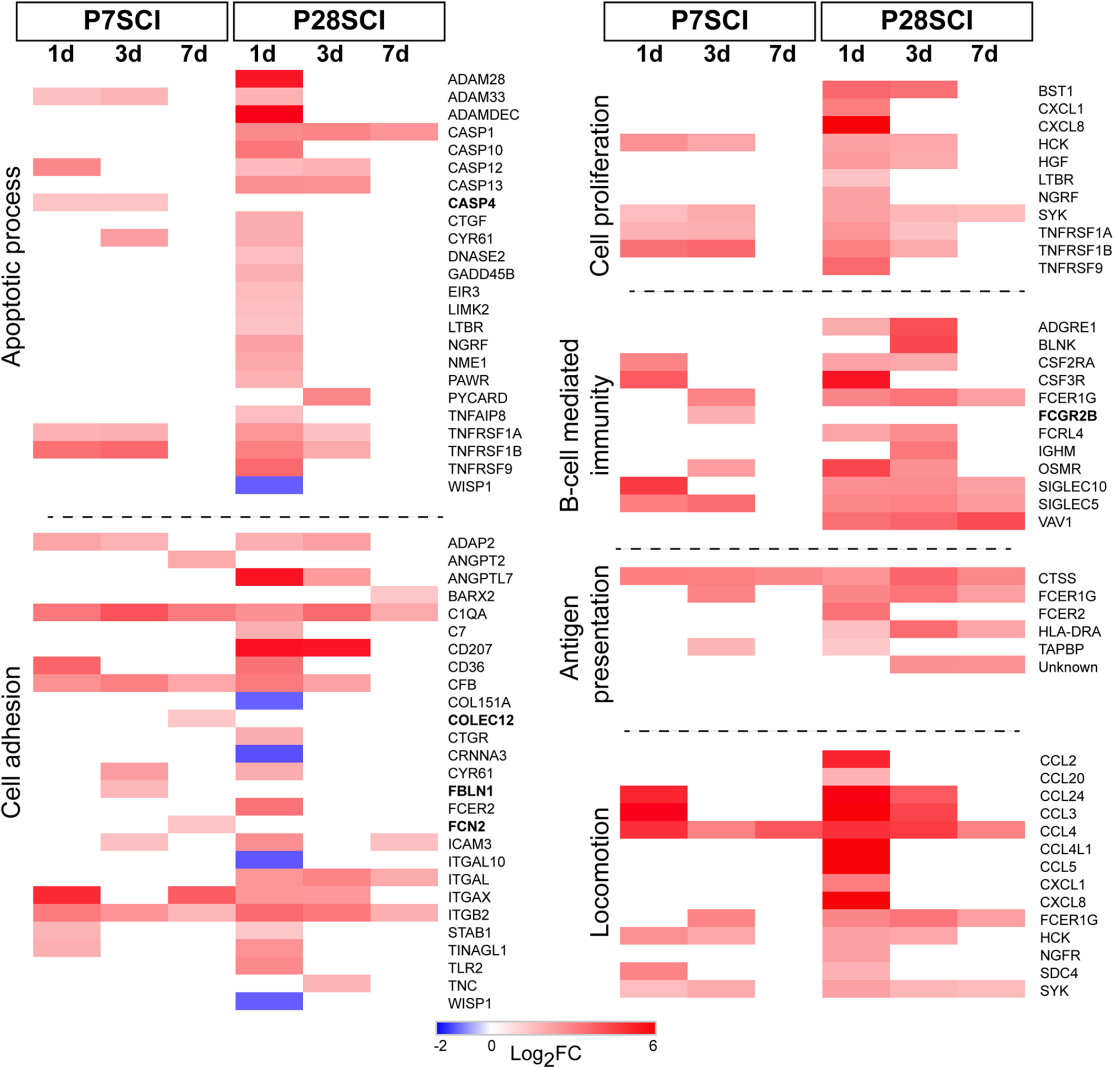
Individual genes classified in key functional categories in only nonregenerating injury groups. The genes that were classified in GO slim categories that were overrepresented only after injury in P28SCI animals are shown along with their fold changes. Bold gene names indicate those that only appear in P7SCI data sets. *Note*: Some genes are classified in multiple groups [Color figure can be viewed at wileyonlinelibrary.com]

Some GO Slim categories were overrepresented at stages after injury in both regenerating and nonregenerating injuries, while others only appeared in one or the other. To examine whether these functional groups comprised similar or different gene‐sets at different time‐points, the genes in our data sets that were classified in several of the GO categories were extracted and shown individually, along with their fold change, in Figures 4 and 5.

Most common elements of the response to injury in both regenerating and nonregenerating cords were the overrepresentation of some functional categories falling under the “Immune response” parent category—“Response to INF‐γ,” “Macrophage activation,” “Complement activation”—and other more general response‐to‐injury categories such as “Cytokine‐mediated signaling pathway” and “Response to stress.” The genes that were contained in these categories are shown in Figure 4.

Within these categories, it was common to find genes that were differentially expressed in both injury phenotypes but at different times after injury. A few genes were differentially expressed earlier in regenerating animals (e.g., *S100A4*, *INPP5D*) or earlier in nonregenerating animals (e.g., *GBP7*, *PTX3*). Some genes were expressed at the same time‐points but were differentially expressed for a longer period of time in regenerating animals (e.g., *MRC1*, *C3*, *CFB*) or for a longer period of time in nonregenerating animals (e.g., *CCL3*, *CCL24*, *IL1B*, *SYK*). It was rare, however, to find genes that were only differentially expressed only in P7SCI data sets (e.g., *COLEC12*, *IFI35*, *STAT1*).

Some functional categories were only overrepresented at time‐points in the nonregenerating (P28SCI) spinal cords. These were generally more wide‐ranging in their classification, encompassing a wider variety of cellular processes including the immune categories “Antigen presentation,” “B‐cell mediated immunity,” as well as cell process categories “Apoptotic process,” “Cell proliferation,” “Cellular adhesion,” and “Locomotion.” These genes are shown in Figure 5.

Individual gene differences were obviously more striking in these categories. Genes classified in the “Apoptotic process” category were rarely found in P7SCI groups, but instead were primarily found in P28SCI + 1d group. Caspase genes (*CASP1*/*10*/*12* and *13*), classical markers of apoptosis, were more strongly regulated in the nonregenerating spinal cords, although *CASP4* was only upregulated in the regenerating (P7SCI) cord. The immune category “Antigen presentation” contained few genes, but evoked a much stronger and longer‐lasting response in the P28 animals, and genes in the “B‐cell mediated immunity” category suggest a much more developed B‐cell response in nonregenerating cord. Genes such as *BLNK*, *VAV1* and *IGHM* are involved with B‐cell development, maturation and activation, none of which was differentially expressed in the regenerating cords. Other categories of interest here included “Cell proliferation” and “Locomotion,” where nonregenerating cords displayed a much stronger chemokine response, and “Cell adhesion,” where the highest number of downregulated genes was found. Genes involved in cell interactions such as *COL15A1*, *CTNNA3*, *ITGA10*, and *WISP1* were all downregulated in P28SCI cords, and were not differentially expressed in the P7SCI cords.

Although GO category overrepresentation revealed that several facets of the immune response (“Macrophage activation,” “Response to INF‐gamma,” and “Complement activation”) were comparable between regenerating and nonregenerating spinal cords, the presence of a wider range of functional categories suggests a much more complex response in the nonregenerating spinal cords, injured at P28.

### 3.3 Regeneration‐associated genes

Although gene ontology analysis is becoming a standard tool for unbiased assessment of functional gene categories in gene‐sets, it still relies on curated databases of assigned gene functions. These gene functions may be based on published material or more complex methods including sequence homology searches. As such, these databases are ever expanding but may not reflect more novel groups of genes thought to contribute to certain processes.

**FIGURE 6:**
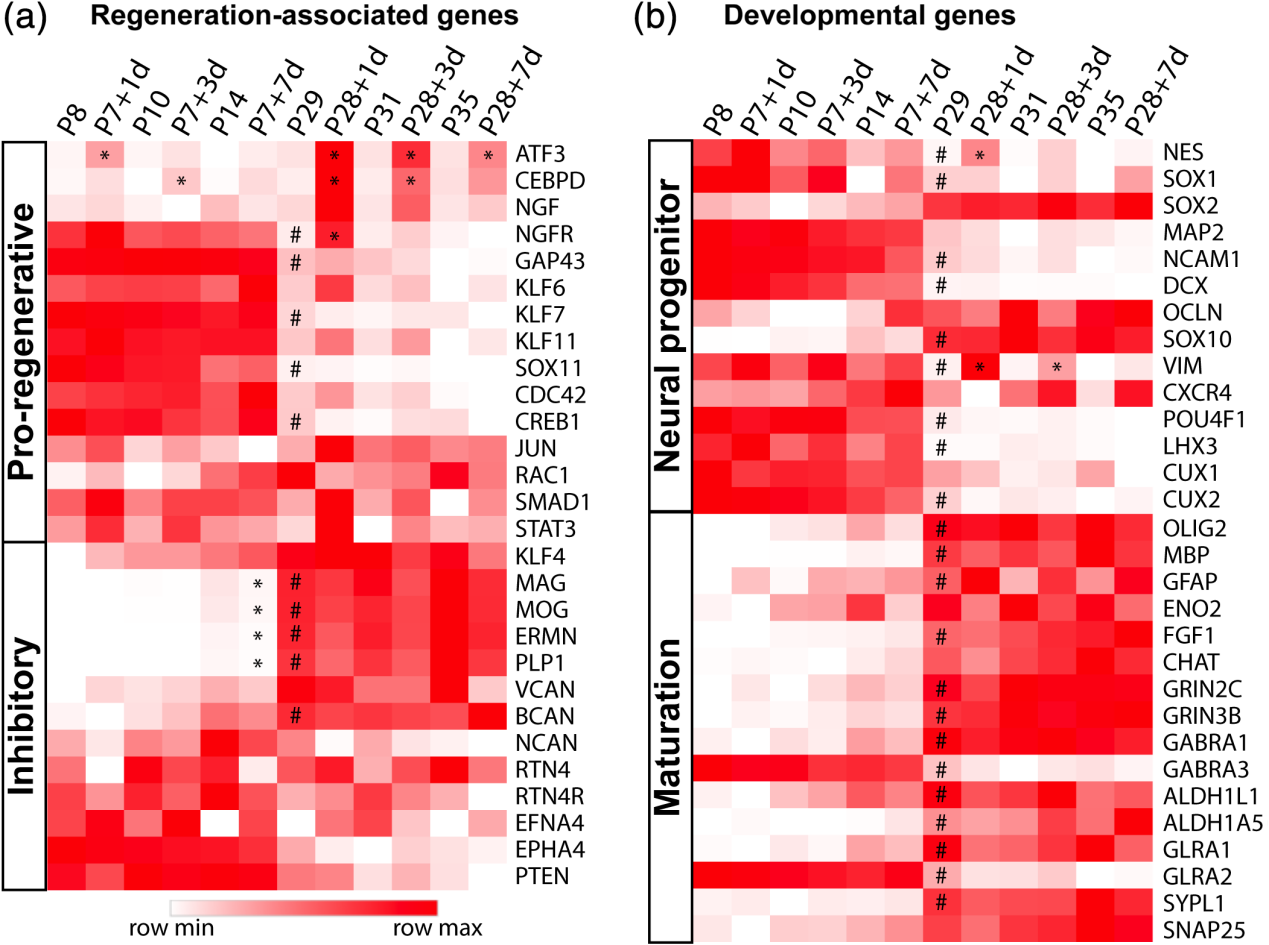
Temporal expression of regeneration‐associated genes (RAGs) and neurodevelopmental genes. FPKM values are shown for representative RAGs, both pro‐ and antiregenerative (a) and developmental (b) genes. Data are expressed as relative expression values across each row. Statistically significant gene expression changes (*Padj ≤* 0.05) for injury induced genes are denoted by *, relative to uninjured age‐matched control; statistically significant gene expression changes (*Padj ≤* 0.05) for developmentally induced genes are denoted by #, relative to P8 control. In both cases differential expression was determined by the DE expression algorithms, under the criteria explained previously [Color figure can be viewed at wileyonlinelibrary.com]

Recently, much research has aimed to identify RAGs. These can include genes that are proregenerative or antiregenerative. In order to assess the role of putative RAGs in our regenerating and nonregenerating spinal cords our transcriptomic data was analyzed for genes known to be either proregenerative or inhibitory genes. Since few of the identified RAGs are differentially expressed in our SCI data sets, normalized expression values (FPKM; resulting from the tuxedo suite analysis package) are shown for all injury groups along with their controls. A selection of these is shown in Figure 6a.

Of the proregenerative genes, the transcription factors *ATF3* and *CEBPD* (Ma & Willis, 2015) were upregulated at some stage after injury at P7SCI; however, these genes were also found to be upregulated after injury at P28SCI to an even greater extent. Other transcription factors such as *KLF6*, *KLF7*, and *SOX11* (Blackmore et al., 2012; Jankowski et al., 2009; Moore et al., 2009) showed no differential expression due to injury, although developmentally these along with others such as *KFL11*, *CDC42*, and *CREB1* (Herman et al., 2018; Ma & Willis, 2015) were expressed at higher levels in the younger, regenerating‐age animals (Figure 6a). Many growth factors thought to be supportive of axonal regeneration are not annotated in the *M. domestica* genome (e.g., *CTNF*) and several others show very low expression (e.g., *BDNF*, *GDNF*; data not shown; Alto et al., 2009; Blesch & Tuszynski, 2003). Interestingly, the NGF receptor (*NGFR*) is upregulated 1 day after injury in P28SCI but not P7SCI animals; however, it is more highly expressed during the regenerative period, but does not change expression due to injury during this period (Figure 6a). Another potent growth‐related factor, *GAP43* (Benowitz & Perrone‐Bizzozero, 1991), shows a similarly elevated expression during the earlier time‐points before its expression decreased as time progressed; it was similarly unaffected by injury. The expression of other putative proregenerative RAGS such as *JUN*, *RAC1*, *SMAD1*, and *STAT3* (Ma & Willis, 2015; Raivich et al., 2004) were unchanged by either development or injury (Figure 6a).

Of the known inhibitory genes, few are affected significantly by injury. The CSPGs brevican, neurocan, and versican (Silver & Miller, 2004; Figure 6a) are not regulated by injury, although versican and brevican's expressions increase during development. The inhibitory ephrin‐A4 (*EFNA4*; Harel & Strittmatter, 2006) and its receptor (*EPHA4*) decrease with time (Figure 6a). The inhibitory transcription factor *PTEN* (Liu et al., 2010) was unaffected by injury, but showed a similar pattern with development. The myelin breakdown product Nogo (*RTN4*) and its receptor (*RTN4R*; Fawcett, 2006) were regulated neither by injury nor development, surprisingly (Figure 6a). Other myelin components, however, such as *MAG*, *ERMN*, *PLP1*, and *MOG* (Fawcett, 2006; McKerracher & Rosen, 2015) all showed dramatic increases in expression with development and were significantly downregulated 7 days after injury at P7 (Figure 6a).

Since these observations potentially point to a loss of regenerative capacity (decreasing expression of proregenerative RAGs during development) and an increase in maturation‐related inhibition (increasing expression of antiregenerative genes during development), we also examined the data for other evidence of a changing environment relating to maturation of the spinal cord. Representative genes are shown in Figure 6b.

Many neural progenitor/precursor genes were significantly downregulated between P8 and P29. These included (but were not limited to) the neural stem cell markers *NES*, and *SOX1* and the neural progenitor genes *NCAM1*, *DCX*, *CUX2*, and *VIM* (Trawczynski, Liu, David, & Fessler, 2019). There were also downregulations of several homeobox genes such as *LHX3* and *POU4F1*, which are involved in motor neuron specification and the development of the sensory nervous system, respectively (Erb et al., 2017; Zou, Li, Klein, & Xiang, 2012). Some neural progenitor genes such as *SOX2*, *SOX10* and *OCLN* (Trawczynski et al., 2019) appeared to increase during development. Interestingly, vimentin and nestin were both upregulated after injury at P28, suggesting that injury, even nonregenerating injuries, are able to induce the expression of some stem cell and neural precursor genes.

Additionally, and unsurprisingly, there were significant developmental upregulations of many markers associated with mature nervous systems. Further evidence of myelination was found in the increased expression of *OLIG2* and *MBP* along with evidence of astrocyte maturation (*GFAP* upregulation). The mature neuronal markers enolase (*ENO2*), *FGF1*, and *CHAT* generally increased expression during development, though only *FGF1* was significantly upregulated at P29. Several neurotransmitter markers were also upregulated during development including those from the glutamate (*GRIN2C*, *GRIN3B)*, GABA (*GABRA1*), dopamine (*ALDH2L1*, *ALDH1A5*) and glycine systems (*GLRA1*, *GLRA2*) and there were similar upregulations of the synaptic components *SYPL1* and *SNAP25*.

### 3.4 Cell‐type–associations of differentially expressed genes

**FIGURE 7:**
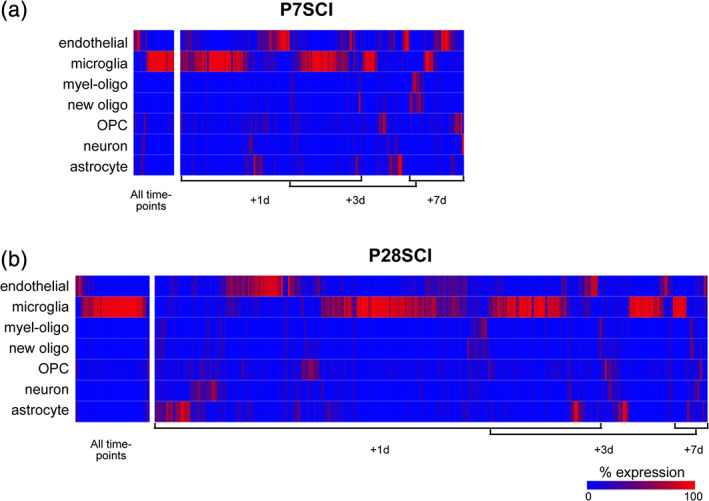
Cell‐type–association analysis showing the predicted cellular localization of the differentially expressed genes identified in this study. Each gene's expression level in each cell type is shown as a percentage of that gene's expression across all cell types. (a) DE genes in P7SCI data sets. (b) DE genes in P28SCI data sets. Cell‐type–specific gene expression predictions were made with reference to published database in Y. Zhang et al. (2014) [Color figure can be viewed at wileyonlinelibrary.com]

We sought to determine which cells are likely to express the genes that we identified in the regenerating and nonregenerating spinal cords, using the published expression data for seven CNS cell‐types available from http://web.stanford.edu/group/barres_lab/brain_rnaseq.html (Y. Zhang et al., 2014). First, the cellular localization data for all genes in the P7SCI and P28SCI data sets was extracted from the available cell‐type transcriptomic database (Y. Zhang et al., 2014); this data was then sorted based on whether the genes were present in single or multiple time‐points, then heatmaps were constructed, and clustered hierarchically (Figure 7).

**FIGURE 8:**
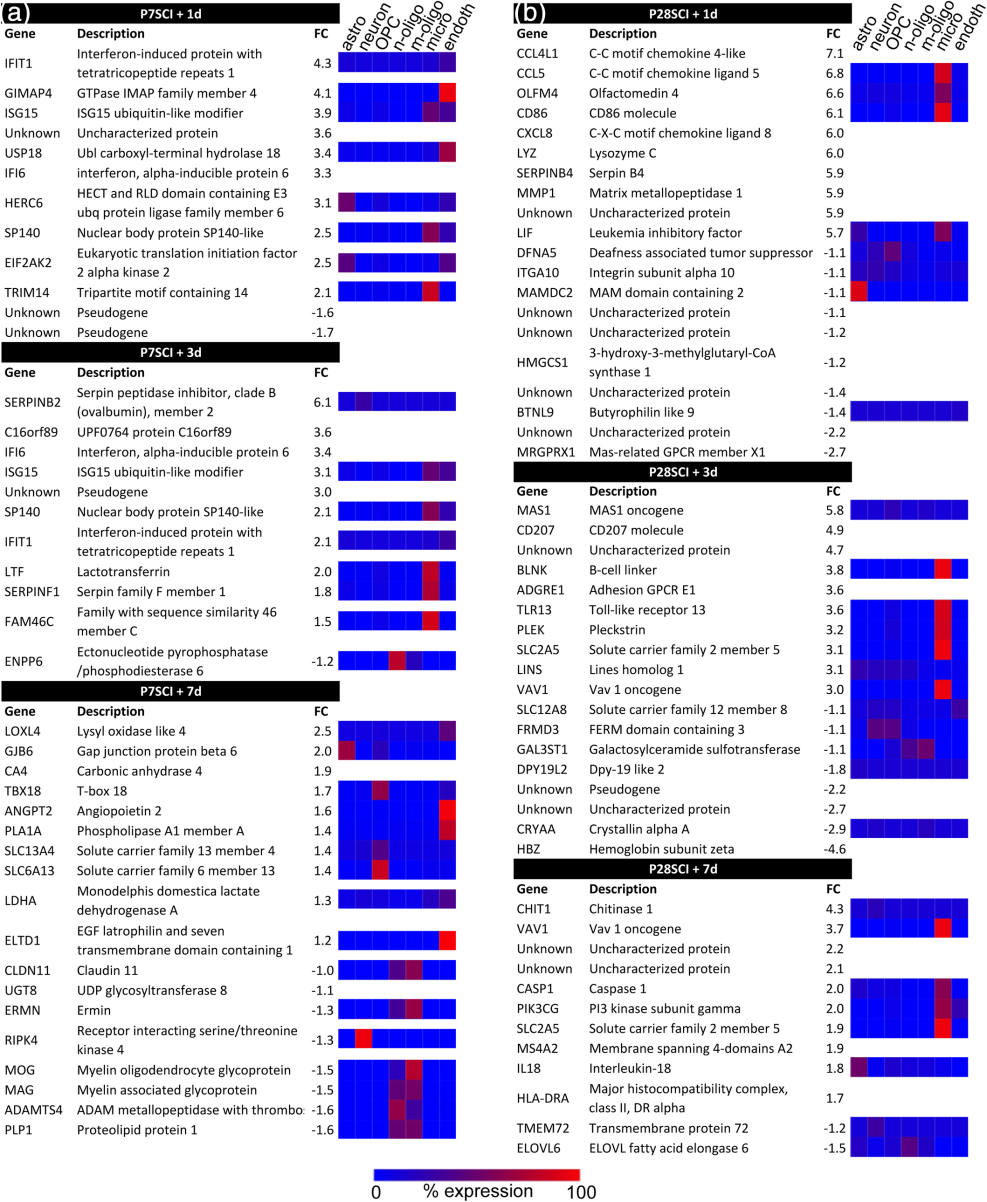
The top unique up‐ and down‐regulated genes and their likely cell source. Genes with the largest fold changes (log2FC) that were uniquely identified in either the P7SCI (a) or P28SCI (b) data sets are shown alongside their cell‐type expression [Color figure can be viewed at wileyonlinelibrary.com]

Next, P7SCI and P28SCI gene lists were subtracted from one another to identify genes that were unique to either regenerating or nonregenerating spinal cords by eliminating all genes that appeared in both P7SCI and P28SCI data sets. Results for the 10 most highly regulated genes uniquely expressed at each time‐point are shown in Figure 8, alongside their likely cellular source. (*Note*: In most cases there were fewer than 10 downregulated genes). A complete list of unique genes differentially expressed in only P7SCI or P28SCI data sets can be found in Appendices 2 and 3, respectively.

Regardless of injury age it was apparent that injury‐associated gene transcripts were expressed most strongly by microglia and endothelial cells (Figure 7a,b), suggesting a strong influence of these cell types in the response to injury regardless of regenerative outcome. Transcripts from these cells were identified across all time‐points after injury in both regenerating and nonregenerating cords, though there were generally fewer at later times after injury, particularly in regenerating (P7SCI) cords. These cell types, particularly microglia, accounted for the majority of injury‐associated gene transcripts in this study and the majority of the most highly upregulated genes found in only P7SCI or P28SCI cords after injury (Figure 8).

There were markedly fewer genes whose expression was biased toward the remaining cell types. Astrocyte clusters appeared acutely in both injury groups, though in P7SCI cords there appeared to be more astrocyte‐associated genes at +3d postinjury whereas a large cluster was found +1d postinjury in the P28SCI tissue. There were strikingly few neuron‐associated genes in either injury group. There was a small cluster at P28SCI + 1d but only a small number of neuron‐associated genes were dispersed across the timeline of P7SCI cords. Genes associated with myelin‐producing cells (OPCs, newly formed oligodendrocytes and myelinating oligodendrocytes) were similarly rare and dispersed with the notable exception of a cluster of genes at P7SCI + 7d (Figure 7a). Many of these oligodendrocyte‐associated genes were, in contrast to most genes identified in this study, downregulated by injury at this time‐point (Figure 8). There was also a group of genes expressing in oligodendrocytes in nonregenerating cords; however, these appeared earlier after injury and comprised a completely different set of (mostly upregulated) genes.

## 4 DISCUSSION

Spinal injury in the gray short‐tailed opossum (*M. domestica*) represents a unique mammalian injury model in which endogenous axonal regeneration can be studied in the mammalian CNS following complete spinal transection and in the complete absence of any external interventions. In the present study we document transcriptomic changes that occur as a result of SCI in postnatal animals where axonal regeneration occurs naturally after injury (injuries made at P7; P7SCI) and those where no regeneration occurs (injuries made at P28; P28SCI).

*M. domestica* is a useful model organism for these studies because its young are born in an extremely altricial state, allowing postnatal access to developmental stages only available in utero in other laboratory animals such as rodents. Complete thoracic spinal cord transection in neonatal opossums during the first week of life is followed by pronounced and rapid axonal regrowth across the lesion site (Fry et al., 2003; Saunders et al., 1995; Wheaton et al., 2011; Wheaton et al., 2013), resulting in recovery of weight‐bearing, coordinated locomotion (Saunders et al., 1998; Wheaton et al., 2011; Wheaton et al., 2013). When these injuries are made at later time‐points the regenerative response diminishes considerably until it ceases entirely by about 1 month of age (Lane et al., 2007; Wheaton et al., 2011). Unlike in other animal models where axon regeneration is studied using nonspinal cord systems (e.g., the peripheral nervous system, cranial nerves) or the partial transections/collateral‐sprouting paradigm, the regeneration seen in *M. domestica* occurs in the CNS after a complete transection and without any external interventions.

Since the response to spinal injury involves the interaction of multiple cell types, both native and infiltrating, injury to the spinal cord is likely to evoke a very complex response. Each of these cell types expresses thousands of genes (e.g., differentiated neurons express more than 10,000; Sammeta, Yu, Bose, & McClintock, 2007) and it is likely that the abundance of many genes in any of these cell types may be affected by injury to the spinal cord. Consequently, studying spinal injury using broad gene identification methods such as RNA‐seq, where hundreds or thousands of differentially expressed genes could be identified can potentially present problems. To address this, in our study a deliberately conservative data set was created, using a stringent set of selection criteria where only those genes whose abundance showed at least a twofold up‐ or downregulation (±log2FC of >1) in expression compared to control and that were identified as such by at least two differential expression pipelines were selected for further study (Hansen et al., 2016). The individual algorithm pipelines we used here produced data sets of different sizes (Figure 1b), leading to the possibility that genes with subtle changes might be missed by our analysis. Although the production of a larger data set (e.g., choosing the algorithm that identified the most differentially expressed genes or relaxing the fold change criteria) could be useful when performing pathway analysis, there is an increased risk of identifying false positives, which may hinder further study into the roles of genes thought to be important. This approach successfully identified broad statistically significant decreases in the expression of neural progenitor and neural stem cell–related genes and increases in expression of markers of spinal cord maturation (myelination, mature neurons and glia) during development (Figure 6b). These gene changes are not surprising given that the cord is developing rapidly during this time resulting in approximately a fivefold increase in number of descending axons (Fry et al., 2003) and an approximate doubling in the cross‐sectional area of the spinal cord during this developmental period (Wheaton et al., 2015). These developmental findings are consistent with our chosen methodology being sufficiently robust and having the capacity to identify biologically relevant and important gene expression changes.

Using this approach more than 500 gene transcripts that robustly changed abundance after spinal injury at one or both injury ages were identified (Figure 2). The number of identified differentially expressed genes decreased over time but each time‐point after injury did contain a substantial portion of genes that were unique and not overlapping with other time‐points. This suggests that, regardless of regenerative outcome, the response time‐course does not simply represent a return to the baseline transcriptome, but that temporally distinct elements are incorporated at later time‐points, as has been shown previously in other regenerating SCI models (Herman et al., 2018). Furthermore, the injuries at the two ages resulted in the transcription of distinct gene‐sets (i.e., not found in the gene‐sets of the other injury age), particularly in the nonregenerating cords where 325 unique genes were identified. This indicates that the loss of regenerative capacity is not simply caused by the induction of weaker regenerative response, but a substantially different response altogether.

### 4.1 Identifying genes and processes involved in the response to injury

So what is actually controlling the regeneration seen in the opossum model? One candidate with the potential to either promote or interfere with regeneration is the immune system, since it is an early responder to trauma, including in the spinal cord where resident microglia become activated and initiate innate and adaptive immune responses (Ankeny & Popovich, 2009). In our study, there are several notable differences in the way the immune system responds to injury at the two different ages.

First, although it might appear that the immune responses following injury were broadly similar since many immune‐related categories were overrepresented regardless of injury age, there are in fact many differences when the individual genes comprising the categories are examined (Figures 4 and 5). Few immune genes were expressed uniquely in regenerating cords and fewer still of these have been described in spinal cord injury. *FBLN1*, an extracellular matrix gene important in cell adhesion and migration, and *FCGR2B*, a structural component of the receptor for the Fc region of immunoglobulin gamma, have both been shown to be upregulated in adult rodent spinal injury (K. Chen et al., 2013; Xu et al., 2015), after which no regeneration occurs. *STAT1* is a transcription factor with putatively negative roles in neural survival after injury (Takagi, Harada, Chiarugi, & Moskowitz, 2002) yet it is upregulated 1 day after injury in regenerating cords, and has been previously identified as being downregulated 1 day after injury in nonregenerating opossum cords (Saunders et al., 2014). Other unique genes associated with the “regenerative” immune response have little evidence of involvement in SCI (e.g., *EIF2AK*, *GIMAP4*, *PLA2D4C*, *IFI35*, *COLEC12*, *IFIT1*, and *RBM43)*. In contrast, nonregenerating injuries resulted in the expression of many unique genes in each immune category and also resulted in the overrepresentation of “B‐cell mediated immunity” and “Antigen processing and presentation,” categories related to the adaptive immune system. In the opossum, the adaptive immune system develops postnatally and the ability to mount B or T cell responses does not fully develop until after the second week of age (Parra et al., 2009; X. Wang, Sharp, & Miller, 2012). The exact role that the adaptive immune system plays in the response to spinal injury is still poorly characterized, especially in the case of B‐cells. B‐cells produce autoantibodies, become autoreactive and persist indefinitely after spinal cord injury (Ankeny, Lucin, Sanders, McGaughy, & Popovich, 2006) and have been shown to be detrimental to anatomical repair and functional recovery (Ankeny & Popovich, 2009). Two genes implicated in B‐cell maturation and activation were identified only in nonregenerating injuries here, *BLNK* and *VAV1*, whose roles are not well characterized in spinal injury, although *VAV1* has been shown to have sustained upregulation in rat SCI up to 8 weeks postinjury (H. Zhang & Wang, 2016) and is required for antigen presentation to T‐cells (Malhotra, Kovats, Zhang, & Coggeshall, 2009). *BLNK* is involved in B‐cell differentiation (Lagresle‐Peyrou et al., 2014) and is over‐expressed in B‐cell lymphoma in the CNS (Akhter et al., 2015) and may also have a role in modulating autoimmunity by regulating the production of IL‐10 (Jin et al., 2013). Nonregenerating injuries also resulted in the differential expression of a greater number of chemokines and the activation of two toll‐like receptors (*TLR2* and *TLR6*; Figure 4) both of which play important roles in mediating activation of the innate and adaptive immune responses in the CNS (Olson & Miller, 2004). Activation of *TLR2* has been shown to activate microglia and result in demyelination and axonal injury in the spinal cord (Kigerl et al., 2007; Popovich et al., 2002).

The more complex immune response occurring in the nonregenerating injuries might result in a greater degree of secondary injury. There was an overrepresentation of genes related to apoptotic processes in nonregenerating cords; in fact these genes were almost entirely limited to differential expression in the nonregenerating spinal cord (Figure 5). Apoptosis can be driven by inflammatory responses by signaling through TNFα receptors, two of which were upregulated in our nonregenerating data set, to activate intracellular caspase cascades (Baker & Reddy, 1998; Keane, Davis, & Dietrich, 2006). The activation of the greater number of apoptosis‐related genes in the nonregenerating cords (including 3 additional caspases and two additional TNFα receptor family genes), possibly resulting from the more complex immune response, is consistent with a more coordinated and potentially more detrimental response to injury resulting in the inhibition of axonal regrowth, as suggested previously (Mladinic et al., 2010). Increased apoptotic potential coinciding with the loss of axonal regeneration has also been shown in chick models of spinal cord injury (Whalley, O'Neill, & Ferretti, 2006).

Second, and most strikingly, is the difference in immune persistence between the two groups. Previous studies in adult spinal cord injury suggest that the immune response may persist indefinitely (Ankeny & Popovich, 2009). We demonstrate here that there was a much more persistent immune response in the nonregenerating animals, with multiple immune related groups overrepresented across all time‐points studied. In contrast, in the regenerating cords, the immune categories and the genes that comprised these categories had almost entirely disappeared by 7 days postinjury, which coincides with the first detectable evidence of axons regrowing across the injury site (Lane et al., 2007). Although our analysis only covered the first week after injury, one could hypothesize that the return to resting immune status may be critical for the promotion of regeneration and that the longer and more complex immune response seen in the nonregenerating cords could be detrimental.

Further studies are therefore required in order to fully elucidate the true nature of this complex immune response, its persistence into adulthood and its role in controlling the transition between regeneration and nonregeneration in this model.

An alternative hypothesis may be that proregenerative signals are able to promote regeneration early in development, and that either these signals are lost as the spinal cord matures or that they are no longer able to produce regeneration later in development due to increased levels of inhibitory or antiregenerative signals or processes.

Several groups have reported that CNS neurons can initiate a regenerative response similar to the successful RAG programs seen in the PNS (for review, see Ma & Willis, 2015) but, in contrast to the RAG response observed in the PNS that lasts until the regenerating axons have found their targets, in the adult mammalian CNS this process is short‐lived and abortive (Mason, Lieberman, & Anderson, 2003; Siebert, Middelton, & Stelzner, 2010). Long‐lasting expression of many RAGs extending into adulthood also follows SCI in the lamprey, in which successful regeneration occurs after injury, further supporting a pertinent role for these molecules in any regenerative response (Herman et al., 2018).

However, in this study few putative RAGs significantly changed expression in response to injury. Indeed, those that did seemed to change their expression more strongly or more persistently in nonregenerating injuries (e.g., *ATF3*, *CEBPD*, *NGFR)*, supporting the idea that a transient but unsuccessful regenerative response might still be mounted after injury but is unable to promote regeneration due to other factors, such as the more prolonged immune response in the nonregenerating cords, a disconnect in the response to injury that has been demonstrated previously (Siebert et al., 2010).

Our analysis appears to support the idea that the spinal cord develops an increasingly inhibitory and complex environment as the cord matures, as was reflected by the broad decline of neural precursor genes and broad increase of genes associated with maturation of the cord (Figure 6b). When injury‐induced gene‐sets were assessed for likely cell source (Figure 7), a surprising observation was that very few differentially expressed genes were shown to be expressed at high levels in neurons, particularly the genes identified in regenerating cords, whereas the majority were expressed at high levels in microglia and endothelial cells, and considerably fewer highly expressed in glial cells (astrocytes, OPCs and oligodendrocytes), suggesting that under the conditions in this study the majority of the transcriptomic response might be occurring extrinsic to the neurons. The expression of proregenerative factors such as *KLF7*, *SOX11*, *NGFR*, *CDC42*, *CREB1*, and *GAP43* decreased significantly during development, a decline that was accompanied by a simultaneous developmental increase in the inhibitory (and extrinsic) factors such as the CSPGs *BCAN*, *VCAN* and the myelin components *MAG*, *MOG*, *ERMN* and *PLP1* (see Figure 6a,b). These increased expression dramatically during development, an observation supported by previous histological work (Ghooray & Martin, 1993). On the other hand, the myelin breakdown product and putative neurite inhibitory molecule Nogo (*RTN4*; M. S. Chen et al., 2000) was unaffected by injury or development, in agreement with previous work in this model (Mladinic et al., 2010; Saunders et al., 2014). Taken together these data are consistent with axon regeneration being negatively mediated by extrinsic factors in the milieu of the cord, rather than any specific, developmentally regulated regenerative response within the neurons themselves.

Most studies of the regeneration in this model have examined regrowth of axons descending from the brainstem. Although it is evident that interneurons also cross the injury site following injury at P7 (Wheaton et al., 2011), no studies have directly examined their development and response to injury in the early stages after injury in this model. But given the nascent developmental state of the cord at P7, the number of interneurons in the cord may be quite small, which could explain why some RAGs were very lowly expressed in our study early in development (e.g., *ATF3*, *BDNF*, *KLF4*, *KLF13*, *NGF*) and few changed expression in response to injury and also why the functional and cell‐type specific analyses were dominated by non‐neuronal factors. Further study into the transcriptome of the brainstem after injury might reveal a different pattern of RAG expression, as shown in the lamprey (Herman et al., 2018). And future studies directly examining the transcriptomes of developing *M. domestica* neurons following injury, either by FACS sorting or single‐cell RNA‐seq, may reveal a more substantive RAG response. Analyses such as these may identify regenerative signals in cell bodies of axotomized axons in the absence of potentially obfuscating influence of the immune system and other inhibitory molecules close to the site of injury, but to date these have not been done.

### 4.2 General conclusions

This study provides a largest survey to date of the transcriptomic changes occurring after injury that may underlie this regenerative response seen after injury in the neonatal opossum.

So what can be concluded from the present study? Is the regenerative response driven by a mechanism that promotes regeneration only early in development but is switched off or downregulated during maturation? Or is there a process activated later in development that prevents regeneration?

Our study potentially suggests the latter and, given the overwhelming number of genes and ontology categories related to non‐neuronal sources and processes, supports the idea that extrinsic (i.e., non‐neuronal) factors may be key in the switch between regenerating and nonregenerating injuries. Our data suggests that following injury in the nonregenerating P28SCI animals a “regenerative” response might still be mounted, as evinced by the fact that many proregenerative RAGs changed expression more strongly after injury at this time‐point. However, the most striking difference that we observed between regenerating and nonregenerating injuries was the development of an enhanced, prolonged and potentially more damaging immune response following injury that incorporated many more elements of the adaptive immune system, persisted throughout the acute period examined here and resulted in the increased expression of many apoptosis‐related genes. No such long‐lasting immune response persisted in the regenerating animals, which may have been a key factor in the success of any regenerative response. We also present evidence of a generalized loss of neurodevelopmental guidance and specification genes and an increase in the expression of several myelin components and other inhibitory genes throughout development. This suggests that regardless of the ability of the neuronal population to mount a regenerative response, there may be a generally more mature and inhibitory spinal cord environment through which they have to grow. Furthermore, no neuronal‐regeneration–related GO categories were overrepresented and the vast majority of genes in this study were most likely predicted to be expressed by glial cells rather than neurons. Taken together these observations point to a powerful influence of developing extrinsic factors potentially acting as a regeneration inhibitor in this model.

## CONFLICT OF INTEREST

No competing interests, financial or otherwise, exist.

### PEER REVIEW

The peer review history for this article is available at https://publons.com/publon/10.1002/cne.24994.

## AUTHOR CONTRIBUTIONS

Benjamin J. Wheaton and Robert D. Miller conceived of and designed the study; Pooja Umale and Benjamin J. Wheaton acquired the data; Benjamin J. Wheaton, Robert D. Miller, Anitha Sundararajan, Johnny Sena, and Faye Schilkey analyzed the data; Benjamin J. Wheaton and Robert D. Miller interpreted the data and wrote the manuscript.

## Supporting information


**Appendix** **1**: Gene expression changes in P7SCI and P28SCI cords. All genes that showed log2FC ≥ 1 in either direction (relative to age‐matched control tissue) with *Padj* ≤ 0.05 and the time‐point(s) in which they appeared. Genes are sorted by Ensembl identifier. Average fold‐change indicates mean value of the significant fold changes as determined by at least two DE algorithms.

**Appendix** **2**: Unique gene expression changes only identified in **P7SCI** data sets. Differentially expressed genes identified in the P28SCI (nonregenerating) data set were subtracted from those appearing in the P7SCI (regenerating) data set, leaving only genes that changed in regenerating cords. Genes are sorted by average fold change.

**Appendix** **3**: Unique gene expression changes only identified in **P28SCI** data sets. Differentially expressed genes identified in the P7SCI (regenerating) data set were subtracted from those appearing in the P28SCI (nonregenerating) data set, leaving only genes that changed in nonregenerating cords. Genes are sorted by average fold change.

## Data Availability

The data that support the findings of this study are openly available in Genbank's (RRID:SCR_004891) Sequence Read Archive at http://www.ncbi.nlm.nih.gov/bioproject/564188, Bioproject accession ID: PRJNA564188.
